# Midwifery and Maternity Care for Single Mothers in Eighteenth-Century Wales

**DOI:** 10.1093/shm/hky092

**Published:** 2018-11-08

**Authors:** Angela Joy Muir

**Keywords:** childbirth, midwifery, old poor law, illegitimacy, Wales

## Abstract

The history of childbirth in England has gained increasing momentum, but no studies have been carried out for Wales, and therefore the nature of childbirth in early modern Wales remains largely unknown. This article seeks to redress this imbalance in two ways: First, by examining Welsh parish, court and ecclesiastical records for evidence of those who attended parturient women. This evidence demonstrates that Welsh midwives were not a homogeneous group who shared a common status and experience, but were a diverse mix of practitioners drawn from a range of socioeconomic backgrounds. Secondly, by assessing the care these practitioners provided to some of the most marginalised in Welsh society: unmarried pregnant women. Parish resources were limited, and poor law provision often covered only what was considered absolutely necessary. Analysis of what was deemed essential for the safe delivery of illegitimate infants provides a revealing glimpse of to the ‘ceremony of childbirth’ in eighteenth-century Wales.

This article explores midwifery in eighteenth-century Wales and the nature of care provided to unmarried pregnant women before, during and after parturition. Evidence of the lived experience of childbirth for most early modern women from the lower orders, both married and unmarried, is scarce. However, parish records, court depositions and ecclesiastical licences do provide evidence of the care available to the poorest women in eighteenth-century Welsh society. Childbirth in early modern and eighteenth-century England has received considerable attention from historians but no similar studies of childbirth or midwifery in early modern Wales exist.^1^ This analysis will therefore begin with an overview of midwifery in Wales within the context of eighteenth-century British childbirth practice. Using parish poor law accounts, ecclesiastical licences and criminal court depositions, this article examines the identities of those who provided maternity care to poorer and unmarried Welsh women, including their socioeconomic backgrounds, skills and experience. After assessing the availability of midwifery services in Wales, the process and rituals sounding childbirth for poorer, unmarried women will be examined using evidence from poor law and quarter session records. Parish resources were limited, and provision to eligible paupers often covered only what was considered absolutely necessary. Analysis of the support provided to unmarried pauper mothers before, during and after birth provides insight into the central features of the ‘ceremony of childbirth’ in eighteenth-century Wales.

The Welsh context of midwifery and childbirth is significant for two key reasons. First, the predominantly rural, remote and scattered nature of the Welsh population throughout the eighteenth century raises questions about the ready availability of the services of individuals skilled and experienced in delivering infants. By examining midwifery in one of the most rural regions of Britain this study serves as a corrective to previous studies of midwifery which have focused on the urban context. At the beginning of the eighteenth century the largest town in Wales was Wrexham, which had an estimated population of between 3,000 and 5,000, followed by smaller market towns such as Carmarthen and Brecon with populations nearer 1,000.^2^ By the end of the century this situation had changed dramatically in south Wales due to the development of mining and industry. However, not all counties were affected by these changes. Throughout the century Wales remained largely rural and agrarian, with small isolated and close-knit settlements brought together by the parish church and connected externally by networks of often poor communication and transport.^3^ Such circumstances bring into question the accessibility of skilled support during parturition for women in these remote communities. However, as this article will demonstrate, the services of at least one woman experienced in delivering babies was readily available to most women across Wales, and every parish appears to have had at least one woman on hand to deliver those in need. Furthermore, the evidence considered here demonstrates that midwives across rural Wales were not a homogeneous group with shared status and experience, but were a diverse mix of practitioners drawn from a range of socioeconomic backgrounds.

The second reason why the Welsh context is significant is due to the high levels of illegitimacy found in some parts of Wales, particularly towards the end of the eighteenth century. Recent studies have established that counties such as Radnorshire experienced a consistently-high illegitimacy ratio that exceeded 10 per cent in the final decades of the eighteenth century, which was more than double the national average in England and represents some of the highest levels in Britain.^4^ Other counties, including Montgomeryshire and Carmarthenshire, experienced ratios between 8 and 10 per cent in the final decades of the century.^5^ Many of the single mothers who bore these children were supported by the parish in the period before, during and immediately after childbirth, while others had less supportive encounters with parish officials. The records of these interactions, as found in poor law documents and quarter sessions records, allow for an interrogation of the processes and rituals surrounding childbirth for poorer, unmarried women. Not all unmarried mothers were destitute, and many were supported during labour by family, friends and employers.^6^ Unfortunately, their experiences are all but lost to us. The records considered here only reveal the treatment of the poorest women and their infants. Although limited, this evidence does provide compelling insight into the complex and often, but not always, fraught experience of bearing a child outside of wedlock in the eighteenth century. As will be seen, single women’s experiences of childbirth depended in large part on their legal status within a community, and on their compliance with parish and legal authorities. Thus, there was no single experience of lying in for unmarried women.

## Midwifery in Wales

The eighteenth century saw some of the most significant changes to childbirth care in Britain before the twentieth century, and included the emergent popularity of the man-midwife, formal training in obstetrics for surgeons, and the establishment of lying-in hospitals in urban areas, particularly in London.^7^ Despite these changes, the vast majority of women of the lower orders of society were attended by female midwives during labour and delivery throughout the century.^8^ Skilled midwives were thus undoubtedly ubiquitous in early modern British society. Their presence can be found in a range of archival sources, and social, cultural and medical historians of early modern England have utilised these for diverse histories of childbirth. Welsh sources have not been considered in any of these studies, and therefore the nature of childbirth in early modern Wales remains largely unknown.

Evidence of midwives in Wales can be found in a wide range of documents, including applications for licences to practice midwifery, Court of Great Sessions and quarter sessions records, parish overseer, churchwarden and vestry accounts, as well as burial records and wills.^9^ Only ten applications for licences to practise midwifery have survived in the Welsh ecclesiastical records held by the National Library of Wales, and found in the collections for the diocese of Bangor and Llandaff.^10^ Many more were likely produced, however the survival rate for such documents in Wales is poor. Court of Great Sessions records have a much greater rate of survival, and midwives appear frequently as witnesses in the approximately 140 infanticide cases included in the Crime and Punishment database between 1730 and 1800. Twelve of these cases have been examined here.^11^ Quarter sessions records from across Wales have also survived to varying degrees and are held in county archives offices. The session rolls of the Montgomery quarter sessions from the 1750s to 1790s have been examined for this study.^12^ Finally, parish overseers of the poor, churchwarden and vestry accounts survive with varying quality for parishes across all Welsh counties, and contain hundreds, if not thousands, of references of payments made to midwives for the delivery of pauper women. For this study, the accounts of 23 parishes across four Welsh counties have been analysed.^13^

The scope and variety of these sources provides ample evidence to suggest that the services of at least one woman experienced in delivering babies was readily available to most women in Wales, and that every parish appears to have had at least one woman on hand to deliver those in need. In general, the state of midwifery in Wales appears to resemble midwifery in provincial England for much of the eighteenth century. By the 1760s, seven lying-in charities had been established in London, but throughout this period there were no formal institutions in Wales, or in most parts of rural England, dedicated to providing maternity care to either married or single women.^14^ Women were attended by midwives in their own homes, or within the community.^15^ The profiles of those who delivered infants in Wales also resemble their English counterparts, and fall loosely into four categories: sworn midwives, midwives who practised without obtaining a formal licence, women who delivered infants but were not identified as ‘midwife’, and male practitioners who were typically identified as man-midwives and surgeons. Women who were simply identified as ‘midwife’ appear most frequently in available records, although it is not always possible to determine if they were formally sworn or not. Likewise, it is difficult to know what, if anything, was signified when a woman was paid for delivering infants without specifically being identified as a midwife. It is highly probable that the distinction between those who paid for a licence and those who did not was negligible, and licences may simply have ratified decisions local women and officials had already made.^16^ These categories will be explored in more detail below, but for the purposes of this study, any woman identified as a midwife, or who was paid for providing the services of a midwife, will be considered as such.

### Becoming a midwife

Throughout the early modern period, midwifery was not a formal trade for most practitioners and the role of midwife was rarely a woman’s only livelihood.^17^ For many, midwifery was a skill one possessed rather than a main social identity.^18^ In rural areas, early modern medical practice in general was typically a part-time occupation for most practitioners due to limited demand.^19^ For female practitioners, the ability to successfully deliver infants and attend to women during labour was a skill learned entirely in the birth room. In London, experienced midwives would formally take on a ‘deputy’ who would learn their craft through what was essentially an apprenticeship. A similar system may have existed in some parts of Wales, although evidence is limited. From the sixteenth century the regulation of midwives was the remit of ecclesiastical authorities, and midwives, as well as physicians and surgeons, were expected to apply to their bishop to obtain a licence. Midwives were also required to be formally sworn into office.^20^ One clause found in several oaths from the diocese of Llandaff states that midwives ‘will not make or assign any deputy or deputies to exercise or occupy under you … but as you shall perfectly know to be of right honest and discrete behaviour …’.^21^ However, beyond this clause, there is no other evidence of a system of deputy midwives in Wales. Doreen Evenden has suggested that these arrangements were unique to London, and thus this clause may simply have been an administrative formality rather than an established practice.^22^ Most midwives in Wales would therefore have learned how to deliver babies through a less formalised system of assisting more-experienced midwives.^23^

In order to obtain a licence, an applicant was expected to provide written testimonies to prove her skills and abilities in the art of midwifery, and her sober and honest character.^24^ Thus, paradoxically, midwives were expected to have successfully delivered numerous women before obtaining the licence that would allow them to legally carry out these duties. The cost of obtaining a licence varied, but could be as high as £2, which would have been a considerable and very likely a prohibitive amount for many women.^25^ Consequently, not all women who practised midwifery held licences. Despite the potential risk of fines and presentments for not obtaining a licence, most women who delivered infants in eighteenth-century Britain were likely not licensed. In Wales, the risk of presentment was minimal, as there were few instances of unlicensed midwives, or any medical practitioners, being prosecuted.^26^ With little chance of ever being called before ecclesiastical officials for practising without a licence, there would have been little incentive to obtain one. However, some midwives did choose to go through the costly process to become licensed. The most detailed evidence of midwives comes from the testimonials accompanying their licensing applications. Despite the poor survival rate, the similarities between these documents and similar English records suggest comparable expectations and responsibilities for midwives. Of the ten women whose oaths or applications survive, eight were described as the ‘wife of’ someone, and one midwife was described as a widow.^27^ The remaining midwife’s marital status was not given, and therefore it is possible she was single, as unmarried midwives in England were not unheard of but were not the norm.^28^ As in England, the majority of Welsh midwives were married women. Reproductive knowledge was restricted, and it was through marriage that women were granted access to the knowledge about the female reproductive body that was central to becoming a successful, skilled midwife.^29^

### Midwives’ reputation

Marital status was not the only requirement for admission into the office of midwife. Of equal, or perhaps more, importance was a woman’s reputation, based on skill and character. Reputation was perhaps a midwife’s, or any practitioner’s, single most valuable trait.^30^ Several of the surviving applications provide testimony attesting to a midwife’s record in successfully attending to women during labour. In Wales, as in England, testimony about a woman’s character and skills came from local officials, such as churchwardens and curates, and from the women she had previously delivered.^31^ In 1753, Wenllian Harry of Whitchurch in Glamorgan was certified to be a ‘sober, modest person and very skilful in the art of midwifery’.^32^ In 1774, Jane Morgan of Llantrisant in Glamorgan was certified by eleven of her neighbours to be ‘a sober modest person of good life and conversation and very skilful in the art of midwifery, and … a proper woman to be licensed to practice the same’.^33^ The 1753 application of Mary Winn of Cardiff provides details about the extent to which some midwives were considered skilled to manage difficult pregnancies and births. Winn’s application contains two personal testimonials from women she had attended who had experienced complications as a result of less-skilled care. Jane Thomas stated that during a miscarriage at four months’ gestation she was attended to by one Jennet Robert, who she described as an ‘unskilful’ midwife, who left her ‘in a most deplorable condition so that [her] life was in the utmost danger’. However, her life was spared after being attended by Winn, whom she described as ‘a woman of great skill and knowledge in her profession’.^34^ Similarly, Wenllian Watkins testified that she, too, had been left in ‘a most deplorable condition’ by the unskilled hand of a midwife named Ann Edward. Upon hearing of her ‘melancholy condition’, Winn was sent for and delivered Wenllian ‘from the jaws of death’.^35^

The socioeconomic status of these midwives is difficult to ascertain, but there are indications to suggest they were of middling status. As the authority of the church courts waned in the eighteenth century, the system of ecclesiastical licensing, which was already weak in Wales, increasingly fell into disuse, particularly after 1750.^36^ Therefore, any licence obtained in the latter half of the century may have been acquired for symbolic rather than practical reasons. Moreover, the fact that they were able to obtain an official licence indicates the means to afford one, and thus licences would have been restricted to the wives of yeoman, artisans and others of similar standing.^37^ The occupation of only one applicant’s husband was given. Elizabeth Anwyl’s husband was described as an innkeeper, an occupation in line with many sworn midwives found in England.^38^ Other indications of the status of these women can be found in the identities of those who vouched for them. All of the surviving testimonials bear the signatures of respected men from the communities, such as churchwardens, portreeves and curates. Many of the women whose names appear on testimonials were able to sign their own names as well, such as Margaret John, Magadalen Jones and Jane Morgan who provided evidence of Elinor Ajax’s skills; Jane Jenkin who certified Margaret David’s skills; and Jane Thomas and Margaret Thomas who vouched for Mary Winn.^39^ Their literacy indicates that these signatories were not of the lower orders of society. If they were part of the midwives’ immediate social networks they were likely to have been of similar social status. It is perhaps not coincidental that one of the only surviving presentments for a midwife accused of practising without a licence is included in a summons for a widow to appear for failing to properly administer her husband’s estate.^40^ In 1755, Elizabeth John Griffith of Anglesey was summoned to appear at the Bangor consistory court ‘to answer to the articles which shall be objected against her touching the … administration of the goods, chattels, rights and credits of Owen Prichards Owen, taylor [sic], her late husband … and also for using or occupying the mistery or calling of a midwife without being thereunto lawfully authorised’.^41^ The wife of a tailor would have been amongst those who could potentially have afforded a licence; however, this may have gone unnoticed until the mismanagement of her late husband’s estate was brought to the attention of the authorities. Her lack of a licence despite her ability to afford one may have helped the ecclesiastical authorities to build a stronger case against her. This presentment was by no means evidence of Elizabeth Griffith’s skills and experience as a midwife, but does provide further evidence of the correlation between licensing and socioeconomic status.

It is possible that many more practising midwives in Wales may have obtained licences, which may have long since been lost or destroyed. These include Margaret Davies who was identified as a ‘sworn midwife’ in a 1742 infanticide trial deposition.^42^ However, most midwives who appear in court and parish records were not identified as having been ‘sworn’, and there is no way of knowing if they were. Whether one was sworn does not appear to have been a significant detail, as it was seldom mentioned in court records when midwives were called upon to provide expert opinions. Court of Great Sessions infanticide records contain numerous examples of women who were called upon to examine the bodies of dead infants and their suspected mothers. These women were often described as ‘practising’ or ‘professional’ midwives with no reference to having been formally sworn. In 1799, when surgeon William Jones was called to examine Hannah John for signs of childbirth, he objected to going without midwife Margaret Williams accompanying him.^43^ In neither William’s nor Margaret’s depositions was she referred to as a ‘sworn’ midwife. Women such as Ellen Davies, who in 1734 was called upon to help ‘ease’ Elizabeth Davies of her afterbirth, was described as ‘by profession a midwife’.^44^ Lettice Lynstone and Anne Edwards, who examined the breasts of infanticide suspect Gwenllian David in 1753, were described as ‘having for several years practised the profession of midwifery’.^45^ Many others, such as Mary Williams and Margaret Williams were described simply as practising midwives.^46^ In some cases, a quantifiable amount of experience appears to have been the most noteworthy credential, such as Dorothy verch Edwards, whose opinion of the body of Jane Griffith’s dead infant was supported by her having practised midwifery for 54 years.^47^ If these midwives were not sworn, it does not appear to have had any bearing on their credibility. Simply having this type of experience, which had been gained in the birthing chamber, but may not have been formally acknowledged by a bishop, may have been enough for a woman to be recognised and respected as a midwife.

It is impossible to determine if the proportion of unsworn midwives in Wales was comparable to England, but as in England, it is evident that a considerable number of midwives would not have taken the oath.^48^ In reality, most would have lacked the means to pay for an official licence. The inability to obtain a licence would not have been a reflection of a midwife’s reputation or capabilities, and the skills and reputations of most would have differed little from those who had licences.^49^ What probably did differentiate sworn from unsworn midwives were individual economic circumstances. Women who worked as midwives came from across the social spectrum, including the lower orders. The above-mentioned Anne Edwards, who examined Gwenllian Davies, was described as the wife of a labourer.^50^ It is doubtful that a labourer’s wife could have afforded the £1 to £2 needed to obtain a licence. Lacking means did not necessarily mean lacking reputation, standing and authority. Despite her lower status, Edwards’ professional skills and opinions were valued enough to be sought by judicial officials as evidence in a murder trial. The same is likely true for all midwives who were called upon in legal proceedings. Countless other midwives were paid by parish officials to aid in the delivery of poorer inhabitants, and often the same women were called upon repeatedly over several years. Mrs Owens of Bettws Cedewain, Mary Goodwyn of Berriew, and Mrs Johnson of Hawarden all appear multiple times for deliveries over a span of several years.^51^ In all instances, when a woman was referred to as a midwife, the English word was always used. The Welsh *bydwraig* never appears in official records; however, official ecclesiastical and secular records were kept in Latin until the 1730s, and English thereafter, so this is not surprising. The use of the title of ‘midwife’ outside of circumstances directly relating to the birth of a child may also indicate a certain level of respect attained after years of practice. The 1700 will of Frances Hughes of Haverfordwest, whose moveable goods were valued at a meagre £12, identified her as a midwife, although there is nothing in the inventory which relates in any way to her occupation.^52^ Frustratingly, very few details are ever given about these women’s status and background, but the fact that they were paid by parishes to perform specific duties, or identified as midwives in other official documents suggest that there was at least some level of recognition of their abilities. There is, of course, evidence of midwives whose skills were considered inferior. The two midwives who had initially attended to Mary Winn’s clients are examples of this.^53^ Additionally, in 1794, Mary Morgan, who was arrested on suspicion of infanticide, told a neighbour that the reason she looked pregnant when she was not was because a midwife who had previously attended to her had mismanaged her delivery, thus leaving her permanently disfigured.^54^ The veracity of these accounts is debatable, but midwives’ skills would certainly have varied. However, it cannot be assumed that there was a direct correlation between holding a licence and possessing greater skills and experiences.

### Fees for midwives’ services

Sworn midwives and their unsworn counterparts from the lower orders would have been called upon to attend to poorer women in their parishes. The midwives’ oath varied throughout the early modern period, and across regions, but one clause appears to have remained consistent: all sworn midwives were required to attend any woman in need, regardless of social status.^55^ Wenllian Harry’s oath bound her to be, ‘ready to help every woman labouring with child, as well the poor as the rich’.^56^ This clause may have discouraged higher status midwives who wanted to be more selective of their clientele from obtaining a licence, as they could earn considerably more for their services by attending women higher up the social hierarchy than they could from serving poorer women.^57^

**Figure 1.**
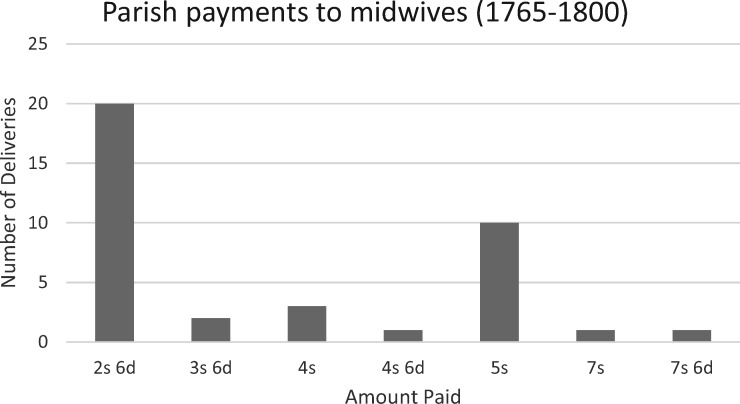
Payments made by parishes to midwives for pauper births, 1765–1800

Parish expenditure on maternity care will be explored further below, but a basic assessment of payments made to midwives by parishes may indicate why few midwives would have obtained licences. From the 1760s parish records in Montgomeryshire, Radnorshire, Denbighshire and Flintshire are full of entries for payments made to midwives for delivering the poor, and every parish for which detailed accounts exist made payments to midwives. These expenses show a clear need for the services of skilled midwives. A sample of 38 payments in seven parishes between 1765 and 1800 made specifically for the act of delivering an infant demonstrates that most midwives employed by a parish were paid 2s 6d per delivery (Figure 1).^58^ Although payments gradually increased over the period, with the highest payments made in 1797 (Castell Caereinion, 7s 6d), and 1800 (Bettws Cedewain, 7s), payments for 2s 6d were made in all four decades. This is consistent with what midwives were paid for delivering pauper women in England.^59^ Midwives who were paid an average of 2s 6d per birth would need to attend to at least 16 births before the cost of a £2 licence was fully amortised. If, as Adrian Wilson has suggested, most midwives had a low caseload of perhaps as few as ten deliveries per year, a licence could represent over a year and a half’s wages. The caseload of most Welsh midwives is indeterminable, but one anecdotal reference from 1845 supports Wilson’s estimate. An epitaph for an ‘experienced and beloved’ midwife named Elizabeth Davies, who died at the age of 81, proclaims that ‘she received more than 300 babies into the world’.^60^ If her career as a midwife spanned 30 years she would have averaged ten deliveries per year. It is likely that some midwives would have delivered significantly more, particularly in towns and larger settlements, but many others would have delivered far fewer. The effort and expense of obtaining a licence would not have been worthwhile for these women, particularly if women practised midwifery as a means of supplementing their household income.^61^ Given the persistent need for the services of a skilled midwife, it would have been in the best interest of ecclesiastical authorities to disregard licensing requirements for those who could not realistically afford one.

For many midwives, the only evidence that they existed at all is when they were mentioned in churchwarden and vestry accounts, when their services were acquired by parish officials. The ambiguity of many entries makes it impossible to map the careers of these women, but most parishes probably had a reserve of two or three trusted women whose services could be called upon.^62^ Very few details accompany entries made for payments to midwives, and often not even a name is given, as countless anonymous entries for ‘paid the midwife’ attest. Between 1765 and 1800 parish officials in Bettws Cedewain made payments for at least 19 pauper births, 12 of which specify payments made for a delivery. Seven of these payments either state ‘paid the midwife’ or ‘paid for delivering’ without identifying who provided these services. Three payments were made to a woman identified only as the wife of Robert Owen, and the remaining two were made to ‘Edward Thomas’s wife’, and to ‘Margaret Thomas, midwife’.^63^ It is possible that these distinctions between those identified as ‘midwife’ and those listed simply by name, or as ‘wife of’ may reflect different levels of experience, where a woman would only be referred to as ‘midwife’ after gaining a reputation for her skills, but there is little evidence to support this. In most cases, this variation appears to carry no overt significance, and may simply relate to the custom of referring to women first and foremost by their marital status. The payment made to an anonymous midwife in 1776 was identical to the payment made to the wife of Edward Thomas in 1781, which would suggest that the services provided by these women were deemed equal.^64^ If midwifery was not considered a formal trade, and if a woman’s main social identity was based on her marital status, the use of the title ‘midwife’ may not have been considered necessary. Moreover, if a woman was paid to deliver a child her status as a midwife for parish officials may have been axiomatic

The differences in amounts parish officials paid to different midwives for their services therefore cannot be said to reflect whether a midwife held a licence, or the level of her skill and experience. Payments to midwives were not standardised, and varied considerably between parishes across Britain; however some patterns in Welsh parish records are evident.^65^ Differing payments appear to reflect varying degrees of difficulties and birth complications, with a basic fee of 2s 6d for uncomplicated pauper births in Wales in the latter half of the eighteenth century. Adrian Wilson has argued that major complications such as obstructions, haemorrhaging and eclampsia were relatively rare. Most births, therefore, would have been relatively uncomplicated, and manageable by most midwives.^66^ However, complications did still occur, and midwives practising for any length of time were likely to encounter these, however infrequently. Higher payments for some pauper births can be attributed to compensation for more difficult or protracted deliveries. In the eighteenth century, as in the present day, the progress of labour was determined by a variety of factors, including the size and shape of the mother’s pelvis, as well as the position, or presentation, of the infant in the birth canal.^67^ Most infants present head-first, which is the easiest presentation to deliver. Breech births are less common but are not rare, and present increased, but not insurmountable difficulties. Most experienced midwives would have been capable of successfully delivering these births.^68^ The most rare and difficult presentations are those in which the infant lies transversely, presenting by the arm, shoulder, back or belly first.^69^ These invariably resulted in an obstructed birth that required more aggressive intervention, and often resulted in the loss of infant life, and risked the mother’s as well.^70^ Other types of complicated births could challenge the skills of midwives. In Berriew in 1794 a midwife and attendant were paid 10s for tending to Mary Rowlands for four days, presumably as a result of a complication which claimed her life, as the parish paid for her funeral three days after the baptism of her illegitimate daughter Ann.^71^ The highest single amount paid to a midwife was in Castell Caereinion in 1797, where an unnamed midwife was paid 7s 6d to attend to Jane Rogers.^72^ The parish spent a total of £9 6s 7½d on her care, maintenance and other related expenses, which included £1 1s paid to a Dr Jones.^73^ The corresponding baptism record provides evidence as to why this level of care was required, as Jane was delivered of twins.^74^

Jane Roger’s lying-in is one of the few documented instances in which a male practitioner attended a pauper woman during her labour. In this case, the male attendant was identified as a doctor, but in other records, these men were identified with the additional label of ‘man-midwife’. Although rare, instances such as this do provide limited evidence of the rise of the man-midwife in Wales. In England, from at least as early as the seventeenth century, male medical practitioners had been called upon to assist in difficult deliveries, but only as a last resort. Prior to the eighteenth century, men played no role in the management of normal childbirth.^75^ This changed dramatically during the eighteenth century with the establishment of obstetrics as a medical specialisation, and its inclusion in formal medical training.^76^ These developments emerged first in London, but soon spread to provincial centres and gradually into rural areas, but their inroads in Wales have never been examined. Historians differ in their interpretations of the reasons behind the rise of the man-midwife. Adrian Wilson and Jean Donnison have argued that fashion played an important role, as upper and middle-class women opted for the more expensive and elite services of a man-midwife, who increasingly usurped the role of female practitioners.^77^ However, others, such as Helen King, have suggested that the shift was supply-led, as medical men increasingly moved into midwifery as other medical fields became overcrowded, thus increasing the availability of their services.^78^ The evidence for Wales unfortunately sheds no light on the tastes of middling and upper class women, or on the numerical prevalence of man-midwives in the country as a whole. Most documented instances relate to practitioners who were called to attend to lower status women, and only in exceptional circumstances. However limited, this evidence does demonstrate that man-midwives were making inroads into Welsh society.

Evidence of man-midwives in Wales is found in many of the same sources where female practitioners are found, such as court records and parish poor law accounts. The earliest mention of man-midwives in the Welsh sources examined here is from an infanticide trial record. In 1765, two men identified as surgeons and man-midwives, named John Kerry of Chester and Honoratus Leigh Thomas of Hawarden, examined the body of a murdered infant.^79^ Roughly ten years later, two other surgeons and man-midwives served as expert witnesses in infanticide trails in Cardiganshire and Denbighshire, and a third appears in a 1795 trial.^80^ Midwifery was not these men’s sole occupation and, like most man-midwives, obstetrics would have been incorporated into their routine practice.^81^ As surgeons first and foremost, their fees for attending to parturient women were much higher than the fees paid to female midwives. For example, a Shropshire surgeon and man-midwife practising in the eighteenth century routinely charged between 10s 6d and £1 1s per delivery.^82^ Man-midwives were not a cost-effective choice for parish poor law officials, and thus only appear in exceptional circumstances. A petition to the overseers of the poor in the parish of Meifod demonstrates how the expense of having to call for a man-midwife could be ruinous for poorer parishioners. In February 1777, Samuel Ward wrote to officials asking them to support his tenant, David Thomas:

His wife has had a very bad time at her lying in, and [he] was obliged to have a man midwife in order to save his wife’s life and the surgeon’s bill is three guineas and will not stay any longer for it which has rendere’d him not able to pay me my rent if the doctor troubles him I must seize for my rent and by that means the children must come on the parish.^83^

That Thomas had to choose between paying his rent and paying for a man-midwife to attend to his wife shows how costly their services were. Man-midwives were therefore only called upon in the most complicated of circumstances. The delivery of Jane Rogers’ twins is one example of this, as is the £1 15s paid to an anonymous doctor by the parish of Llanfihangel-yng-Ngwynfa in 1797 for the delivery of Robert Ellis’ wife. In this case, the parish also paid for a coffin, shroud and ale for her funeral, which is indicative of a fatal complication.^84^

As male practitioners were called upon only in emergency situations for pauper deliveries, they would have been sent for only after a woman had been labouring under the care of a midwife for some time.^85^ The male practitioners who attended Jane Rogers and Robert Ellis’s wife would therefore not have been their primary attendants. The midwives attending these women in the first instance were likely the individuals who summoned help when the severity of the situations became apparent. Thus, it was essential for surgeons and man-midwives to be known and respected by female midwives.^86^ Evidence from infanticide trials suggests that this type of professional respect was reciprocated. Surgeon William Jones’ insistence that he be accompanied by midwife Margaret Williams is indicative of their working relationship.^87^ It does not follow that all midwives would have automatically respected all surgeons and man-midwives, or vice versa. However, a professional relationship clearly did exist between some of the male and female practitioners who attended to pauper deliveries.

This evidence only speaks to the care of the poorest women in Welsh society, as evidence of those who attended women higher up the socioeconomic ladder in Wales is scarce. Female midwives would have predominated throughout Wales during the eighteenth century, with the services of male practitioners gradually becoming more available as the century progressed. Man-midwives could have been increasingly hired to attend to the middling and upper classes not only for more complicated births, but also for those who preferred their services. The fact that they were available to attend to poorer women at all, and to provide testimony in criminal proceedings, shows that they did have a presence in Wales. It is therefore clear that a diverse range of skilled practitioners were available to at least some degree to attend to all parturient women in Wales. However, the treatment and care women received during childbirth, and in the periods immediately before and after, could vary considerably, especially for women who were not only poor and pregnant, but also unmarried.

## The Ceremony of Childbirth for Unmarried Women

In eighteenth-century Britain, as in all periods, childbirth was more than just the physical act of delivering a child: it was a social event and rite of passage which carried immense social and cultural significance. Birth involved a series of prescribed rituals and participants: the birth room was to be prepared in particular ways, a mother was to be confined for the duration of her lying-in, which included the time leading up to and following delivery; certain individuals were to be excluded and others expected to be in attendance.^88^ These elements served the interests of not only the mother and the infant, but also the community. However, these interests could at times be conflicting, particularly when mothers were poor and unmarried.^89^ Women’s access to each of the prescribed elements varied depending on a range of factors beyond marital status, including financial means and right of settlement in a parish. For married women of means, birth could be a period of celebration which conformed to prescribed expectations, and where a new life was welcomed into the wider community. However, if a child was born to a single mother who lacked the means to support herself and her child, the community could be far less welcoming. In these circumstances celebrations could be replaced with conflict and anxiety. Yet even for unmarried women, the experience of childbirth could vary immensely, and the evidence from Wales demonstrates that not all single mothers were treated with the same level of hostility.

Our understanding of the rituals surrounding the process of giving birth in early modern Britain are drawn almost exclusively from prescriptive literature and from autobiographical accounts from the middling sort and elites.^90^ These accounts portray a frequently female-centred ritual with clearly defined rules, roles and expectations. Adrian Wilson’s assessment of what he has dubbed, ‘the ceremony of childbirth’ portrays the rituals of childbirth as collective female celebrations and social occasions, where women of the community gathered to witness and support the delivery of one of their neighbours. This was a social occasion which also served to reinforce social relationships and hierarchies.^91^ However, Laura Gowing has argued that the birthing chamber was not necessarily a supportive space, and the social experience of childbirth for many women, married or single, could be characterised by tension and fear.^92^ Unfortunately, available records from Wales do little to reveal the dynamics of the birth room in the eighteenth century. However, poor law and quarter sessions records do provide compelling evidence of the types of care provided to some poorer women, both married and unmarried, which hint at some of the tensions which could be present. Comparison of the support provided to poor unmarried parturient women with that of their married counterparts, and to the idealised ritual of childbirth in eighteenth-century Britain provides further insight into the complex attitudes towards, and anxieties surrounding, childbirth outside of marriage.

In its most ideal form, the ceremony of childbirth in eighteenth-century Britain involved the confinement of a woman who was near her time of delivery in a chamber lit by candles, warmed by a fire, and sealed off from outside air and light.^93^ All men, including the expectant baby’s father, were to be excluded from this space, although this gradually changed with the rise of the man-midwife. The mother was to be attended by a midwife whose authority dominated the birth room, and a group of adult female family and neighbours known as the ‘gossips’ who provided assistance. After delivery, a woman’s confinement would continue through the period known as ‘lying-in’ during which time she would stay in bed for up to one month to rest and recover from labour. After her period of lying-in, the mother would be welcomed back into the community through the ritual of churching, which could correspond with the baptism of her child. The reality for many women would have come nowhere near this expectation. Not all women would have had access to a separate room where she could withdraw, or have the means to acquire supplies such as candles and curtains, and many would not have been able to retire from their routine domestic tasks for an entire month.^94^ However, it is likely that most women would have adapted many of these ritual elements according to their means.^95^ Laura Gowing has argued that many of these elements would have been modified even further for unmarried mothers. For example, the neighbourliness of gossips and the supportive role of midwives could be transformed into punishing interrogation.^96^ However, many poor, unmarried women did receive basic elements of support nonetheless. By breaking the ceremony of childbirth down into its component parts and comparing recorded evidence of pauper experiences of the space and place of birth, the period of lying-in and its related rituals, and those who attended poor and unmarried women in Wales, it becomes clear that the experience of childbirth for unmarried mothers varied widely.

Where a woman gave birth was important in two ways: as set out above, the physical space, or birthing chamber, in which the birth took place was expected to conform to certain criteria which informed who and what should be included or excluded. However, the geographic location of the birth was also important, especially for poorer women. Before the poor-law reforms of the nineteenth century, a child gained legal settlement in the parish in which it was born.^97^ An Act from 1662 gave overseers of the poor and justices of the peace the authority to remove those who did not have the legal right of settlement to their last known place of settlement.^98^ Poor law accounts demonstrate that parish officials spent a considerable amount of time and money ensuring they were not burdened by the expense of supporting pauper children born within their bounds. In 1738, overseers in Trefeglwys presented an unmarried pregnant woman named Anne Bamford to a justice of the peace who ordered her to be removed to the parish of Newtown at a cost of 17s.^99^ In 1770 the parish of Kerry spent 6s 1d to remove a ‘vagrant girl that was with child’ to the parish of Bettws Cedewain approximately 7 miles away.^100^ These women were removed because they were poor and pregnant, and therefore posed a long-term financial risk to parishes. Both married and unmarried pregnant paupers could be removed from a parish, although most entries in Welsh records relate to the removal of single pregnant women. Sympathetic parishioners did occasionally take pregnant strangers in, either because of a personal connection to them or simply out of charity. Harbouring an unmarried pregnant woman, however, was a punishable offence, and authorities did act against those who took women in.^101^ For example, in 1767 an overseer in Manafon ordered a parishioner to ‘turn a woman that was big with child out of his house’.^102^ This practice meant that a poor, unmarried pregnant women could find herself on the receiving end of hostility if she was visibly pregnant or found herself in labour in a parish where she did not officially belong.

For a poor, single woman who lacked settlement, going into labour was undoubtedly an anxious and distressing time. Fragments of evidence of what this experience may have been like can be found in court records. In 1750, a recently-delivered single woman named Elizabeth Tomley was examined by the Montgomeryshire Quarters Sessions regarding her settlement.^103^ Her account provides a rare glimpse of how harrowing some women’s deliveries could be. Elizabeth had previously worked as a covenant servant in the parish of Llansanffraid-ym-Mechain, but after her period of employment ended she ‘wandered about the country following one Evan Rosser Russell a quack doctor chiefly residing in Llanfair’, and she became pregnant by him. Early one morning, Elizabeth found herself in labour in the parish of Llanfair. Because she did not have legal settlement, ‘the parishioners being alarmed thereat and fearing least the child should be born therein, and thereby gain a settlement, she could gain no admittance into any house but lay in that condition under the open air’. Elizabeth continued in that state, unaided, throughout the day until the high constable came to her and ‘used many threatening words to cause her to be gone’. Being in labour, Elizabeth was not able to make her own way out of the parish, and the constable ordered his servant to ‘carry her by force on horseback towards the New bridges’ in the neighbouring county of Meifod. Once there, she was set down and left to ‘crawl with great difficulty and anguish’ until she was finally taken in. That same evening she was delivered of a son by a midwife provided by parish overseers, and both Elizabeth and her child were then maintained for an undetermined period of time by the parish of Meifod.

Elizabeth’s experience bears striking resemblance to the treatment of poor, unmarried women in early modern England.^104^ In her case, she was safely delivered by a midwife, but not all women were so lucky. Other single women, either by choice or necessity, did not or could not acquire similar support. In 1792, a pregnant servant named Mary Powell was removed from the parish of Clyro, Radnorshire to Llanhamlach, Breconshire fifteen miles away. Soon after, she gave birth to an infant girl in a cowshed.^105^ Unsurprisingly, Mary’s child did not survive, and Mary was suspected of having murdered her daughter, although she was not indicted. The threat of this type of treatment could have motivated many poor, single pregnant women to conceal their pregnancies and take extreme measures, but could also put women in precarious circumstances which jeopardised their lives and the lives of infants. Even if a woman without settlement was safely delivered of her child by a midwife, lying-in as a stranger could still carry consequences. When Sarah Owen fell into labour in Denbighshire in 1788 she sought help at the house of a blacksmith, where she was delivered of a son by a midwife. Fearing the repercussions from parish officials for having allowed an unmarried stranger to lay in at his house, the blacksmith escorted Sarah out of the parish. Her infant was later found dead and buried in a field.^106^ Abandonment and murder represent the most desperate measures to which mothers resorted if they were unable to access any form of maternity support. However, the aggressive reluctance on the part of some parishes to provide care to unmarried parturient women can be seen as equally negligent or violent, and often resulted in identical outcomes. After officials paid 15s for the removal Estar Lowe from the parish of Llandiam in 1753, officials then paid an additional 1s towards her infant’s funeral.^107^ How far the actions of Llandinam officials contributed to the death of her child is unclear, but her removal could not have improved the infant’s survival chances.

Not all unmarried pregnant women were moved about in this manner. Many other women went into labour in their parish of legal settlement, either because they were already present in their parish, or because they returned before going into labour. When this happened officials had a legal requirement to provide care, and parish poor law accounts are full of examples of this in practice. Although far less tantalising and detailed than examinations and depositions of women who were displaced, this evidence is equally telling. It is also more abundant, which suggests that violent displacement was not the norm. As in the case of Elizabeth Tomley, removal could involve simply dumping an unwanted individual across parish boundaries, however it could also be a two-way process where officials from one parish would remove and officials in the parish of legal settlement would receive. This could come at a sizeable cost for the parish of settlement. When justices of the peace ordered Eliza Evans back to Trefeglwys, her parish of settlement, the overseers of Trefeglwys paid £1 5s 4d on her account, which included the cost of delivering her of her child.^108^ It is difficult to imagine Eliza being warmly welcomed back to her home parish, but she was brought back and provided for nonetheless.

The support Eliza was given during her delivery can be understood as a modified version of the ceremony of childbirth experienced by other women in her community.^109^ If a single woman could not make her own arrangements, or did not have a family to return to, the parish would provide a suitable place for lying-in.^110^ Young single women could lay-in at the home of her parents, such as the daughter of Thomas Williams, whose father was paid 16s for being delivered and maintained in his home.^111^ Overseers frequently made payments to parishioners who lodged poorer married and unmarried pregnant women during their deliveries.^112^ Unmarried women could be maintained in the home of a parish official, such as in Berriew in 1780, when an overseer received payment for lodging a single woman named Catherine Stokes in his house when she delivered her child.^113^ Women could also be maintained in the homes of other neighbours, such as David Davies who kept Mary Calcot in his home in Llangadfan when she was brought to bed in 1770, or Ann Russel of the same parish who took in a single woman named Jane Davies when she was delivered in 1764.^114^ Depending on the wealth of the person who took them in, unmarried women who gave birth in the home of a neighbour may not have had a separate room dedicated to her confinement. However, many married women who gave birth at home would not have a separate room at their disposal either.^115^ What is significant, however, is that when under the care of the parish, women were provided with a space to give birth, and often this care resembled the care provided to married pauper women, as evidenced by payments made for married women during lying-in which covered the costs of midwives and lodging.^116^

In these circumstances, single pregnant women were not being harboured, but were officially lodged and cared for by their parish. What single women experienced when they were tended to in the homes of neighbours or parish officials cannot be known, but it was possibly not entirely supportive or positive. The taking in of unmarried parturient women by parish officials could serve purposes that went beyond basic Christian charity, as this practice could have operated as an extension of the bodily surveillance of unmarried pregnant women.^117^ The maintenance of unmarried parturient women could have been as much about the provision of care as it was about preventing further deviance, such as concealment and infanticide.^118^ The precariousness and uncertainty of childbirth for all women meant that witnesses were required, and from the seventeenth century women were legally prohibited from giving birth alone.^119^ Birth attendants did support mothers and midwives, but could also provide vital evidence about what took place in the birthing chamber, such as witnessing that a still-born child had indeed been born dead and was not a victim of infanticide.^120^ A birth which took place without witnesses raised suspicions, especially if it resulted in a dead child.^121^ Birth attendants were therefore an essential part of the ceremony of childbirth for all women, and Welsh officials ensured they were present for the deliveries of unmarried mothers. In 1787 the parish of Bettws Cedewain provided a midwife and other attendants for the delivery of Catharine Morris.^122^ In 1797, Castell Caereinion officials paid a midwife and an attendant named Anne Evans to attend to Jane Rogers.^123^ In the only example of the word used in the Welsh Records considered here, officials in Guilsfield paid 1s ‘for the gossips’ who attended the delivery of Jane Rowlands in 1769.^124^ Gossips could be friends or family of a mother, but they also had a duty to the community.^125^ When they were provided by the parish, it is possible that their loyalty lay in serving the interests of the community rather than the single mother, and they could have been expected to serve as witnesses and discuss publicly what took place in the birth room. Welsh parish records do not provide details about who these women were, what their relationship to the mother or role in the birth room was, but it is possible that these women provided support to the mother and midwife while also serving as witnesses in the interests of the wider community.

Historians have also argued that, for unmarried mothers, birth attendants served an additional purpose. As a respected member of the community, a midwife was required not only to safely deliver a woman, but also to uphold the interest of the parish.^126^ Numerous historians have suggested that midwives would threaten to withdraw their services during the most extreme moments of labour in order to force an unmarried mother to name the father of her child.^127^ The female attendants present could assist in this punishing treatment, and bear witness to any confessions that resulted. Evidence of this practice can be found in some parts of Britain. A filiation order, which was a legal document that formally identified a father, from Manchester in the 1730s contained such evidence:

Jane Charnock of Manchester aforesaid widow examined upon her oath before us saith that she was present when the said Mary Gaskell was in extremity of labour of Phoebe Warburton the aforesaid bastard child and did officiate as midwife and that the said Mary Gaskell did then confess and declare that the above mentioned Charles Warburton is the only father thereof.^128^

However, this practice does not appear in all regions, and is absent from filiation orders and bastardy bonds from eighteenth-century Wales.^129^ The midwives’ oath varied, and in some instances, did include a clause requiring midwives to extract information about paternity, but often this was not the case.^130^ The surviving Welsh oaths do contain a clause relating to paternal identification, but emphasised their duty to not extract a false confession. Sworn Welsh midwives were required not to force women ‘to name or put any other person to be the father of her child, but only him who is the very father therefore’.^131^ Thus, although sworn Welsh midwives were authorised to ascertain a father’s identity, they were expected to take caution not to extract a false confession under duress, and were not explicitly bound to obtain the identity of a father. Secular authorities had no statutory power to force midwives to extract information about paternity, and there is limited evidence of midwives in Wales presenting evidence relating to paternity before Justices of the Peace.^132^ Welsh records indicate that most unmarried mothers formally identified the fathers of their children prior to their birth, meaning there was less need for such punishment. A survey of 197 eighteenth-century bastardy bonds from six parishes in Montgomeryshire and Radnorshire shows that over half of all bonds (56 per cent) were drawn up while a woman was still ‘with child’ rather than being ‘lately delivered’ or ‘now delivered’.^133^ As argued elsewhere, higher levels of illegitimate paternal identification may have been due to communal awareness of conjugal courtships which were thwarted prior to marriage because of adverse economic circumstances.^134^ This suggests that there may have been no need for midwives and gossips to forcefully extract, or threaten to extract, information about paternity during a single woman’s delivery, as the father’s identity was already established, either formally or informally, by the time a woman went into labour.^135^ The midwives and gossips attending unmarried pregnant women in Wales would therefore have served the dual role of witnesses and carers, as they would have for any other birth. However, they would have attended to their duties with varying degrees of compassion or hostility depending on their relationship with the mother, and their opinions surrounding her circumstances.

One final way in which the provision of care towards unmarried parturient women can be explored is through their support during the lying-in period over the days and weeks following delivery. Under ideal circumstances, a woman would lay-in for the period of one month following the birth of her child, and would then be welcomed back into the parish community through churching. With the exception of two married women who were churched in the parish of Gladestry in 1696 there is no evidence of churching in any of the Welsh records considered here. However, there is evidence of married and unmarried pauper women being provided a lying-in period. During this time, it was expected that a new mother would remain confined to bed, and would refrain from work, going to church and, for married women, engaging in sexual activities with her husband.^136^ It would have been impractical for many women to remain confined for an entire month, and many single mothers in particular would have lacked the means to allow them to lay-in for so long.^137^ However, Welsh parish records do show that provisions were made for unmarried pauper women to lay-in. Although it was not always for a month, it was often at least as long as the period provided to their married counterparts. In 1788 parish overseers in Llanarmon Dyffrin Ceiriog paid 2s 6d towards the lying-in expenses of Griffith Jones’ wife, which was the same amount paid to unmarried women of the parish during their lying-in.^138^ The duration of her lying-in period is not specified, but many unmarried women in other parishes lay-in for anywhere from one week, such as Catherine Morris of Bettws Cedewain and Elinor Nicklas of Guilsfield, to several weeks or months.^139^ At the furthest extreme is the thirteen-week lying-in period of Jane Jones of Castell Caereinion in 1795, at a cost of 16s, when she was delivered of her second child outside of wedlock.^140^ The reason for this extended lying-in period is not given, but the fact that parish officials supported her for this length of time is significant. In 1772, Elinor Jones of Llandrindod was similarly supported for a lying-in period of six weeks at a cost of 1s per week, followed by an additional period of four weeks at 5s per week.^141^ Jane and Elinor likely both suffered complications that necessitated extended lying-in periods, and parishes supported them in this. Other single women, such as Gwen Thomas of Meifod, were allotted the prescribed lying-in month.^142^ Like many Welsh single women, Gwen’s child was put out to nurse, which meant she would not have been prevented by child care duties from returning to any employment she may have held before lying-in. Despite this, Gwen was still allotted a full month’s lying-in. What is significant is that single women were not only allotted a period of lying-in, but that parishes appear to have supported married and unmarried pauper women in similar ways.

## Conclusion

It is difficult, if not impossible, to know if single women who were in receipt of parish support during their deliveries were treated with compassion or with contempt. For many unmarried women, the experience of the later stages of pregnancy and parturition may not have been a period of celebration, but neither was it inevitably a period characterised by conflict and hostility. Women’s experiences would have varied depending on their reputations and standing in the community, and the circumstances surrounding the conception of their child. Many women likely did experience hostility from those who attended to them, but there is evidence of benevolence in the treatment of some unmarried mothers and infants as well. If an unmarried mother was in her parish of settlement during the later stages of her pregnancy, and if she conformed to expectations by making her pregnancy known, and cooperated with officials about the identity of her child’s father, then she was likely afforded the same care provided to married pauper women. However, if she resisted the authority of her parish and her community, or if she fell into labour in a parish in which she did not belong, her experience would have been far more conflicted. Marital status was therefore only one of several factors which influenced the care provided to women. Thus, there was no single experience of childbirth for unmarried mothers. This evidence necessitates a reconsideration of the role of communities in illegitimate childbirths to further examine the diverse, complex and nuanced relationships between neighbours, authorities and single mothers, as these encounters were not universally characterised by hostility and conflict.

The evidence examined here is exceptional in that it relates only to the poorest members of society who were in receipt of parish support. Moreover, most of these records relate to women who bore children outside of wedlock, which again, were the exception rather than the norm. However, the management of illegitimacy and poverty in the eighteenth century has produced some of the only evidence of the experience of childbirth for women from the lower orders. Although this evidence relates only to the poorest women in eighteenth-century Welsh society, the fact that so many of the elements of the ceremony of childbirth were observed in unmarried pauper births strongly suggests that these experiences would have been replicated across Welsh society, and British society more broadly. The care provided to women was based upon what was considered necessary for a ‘proper’ birth experience. If even the poorest unmarried women were provided with the services of a midwife, and allowed a period of recovery afterwards, it is likely that all women in Welsh society had access to this type of care. Regardless of status, childbirth would have been an anxious and difficult time for all women in eighteenth-century Britain.^143^ Welsh women, both married and unmarried, would have been tended to in similar ways to women in other parts of the British Isles. Despite its remoteness, most women in eighteenth-century Wales would have had access to support from an experienced midwife and, as the century progressed, the services of male practitioners as well. Thus, the evidence considered here of the availability of midwives services, and the nature of care provided is revealing not only of many pauper women’s experiences, but most women’s experiences in eighteenth-century Britain to at least some degree.

## Funding

This work was supported by the Wellcome Trust under Grant number WT104885MA; and Social Sciences and Humanities Research Council of Canada under Grant number 752-2015-0033. Research for this article was undertaken at the University of Exeter.

## Footnotes

1 Leah Astbury, ‘Being Well, Looking Ill: Childbirth and the Return to Health in Seventeenth-Century England’, *Social History of Medicine*, 2017, 20, 500–19; Lisa Forman Cody, *Birthing the Nation: Sex, Science and the Conception of Eighteenth-Century Britain* (Oxford: Oxford University Press, 2005); Doreen Evenden, *The Midwives of Seventeenth-Century London* (Cambridge: Cambridge University Press, 2000); Valerie Fildes, ed., *Women as Mothers in Pre-Industrial England*, 2013 edn (London: Routledge, 1990); Mary Fissell, *Vernacular Bodies: The Politics of Reproduction in Early Modern England* (Oxford: Oxford University Press, 2007); Laura Gowing, *Common Bodies: Women, Touch and Power in Seventeenth-Century England* (New Haven, CT and London: Yale University Press, 2003); Samantha Williams, ‘Experience of Pregnancy and Childbirth for Unmarried Mothers in London, 1760–1866’, *Women’s History Review*, 2011, 20, 67–86.

2 Geraint H. Jenkins, *The Foundations of Modern Wales, 1642**–**1780* (Oxford, 1993), 88.

3 Jenkins, *Foundations*, 87.

4 Angela Muir, ‘Courtship, Sex and Poverty: Illegitimacy in Eighteenth-Century Wales’, *Social History* 43 (2018), 60–2.

5 Muir, ‘Courtship, Sex and Poverty’, 62; Anna Brueton, ‘Illegitimacy in South Wales 1660–1870’ (unpublished doctoral thesis, Leicester, 2015), 48.

6 Tanya Evans, *Unfortunate Objects: Lone Mothers in Eighteenth-Century London* (Basingstoke: Palgrave Macmillan, 2005), 145–72.

7 Helen King, ‘Midwifery, 1700–1800: The Man-Midwife as Competitor’ in Anne Borsay and Billie Hunter, eds, *Nursing & Midwifery in Britain Since 1700* (Basingstoke: Palgrave, 2012), 107–27; Jean Donnison, *Midwives and Medical Men: A History of the Struggle for the Control of Childbirth* (New Barnett: Historical Publications, 1988), 37; Evans, *Unfortunate Objects*, 145–6; Adrian Wilson, *The Making of Man-Midwifery: Childbirth in England, 1660**–**1770* (London, UCL, 1995).

8 Lisa Forman Cody, ‘The Body in Birth and Death’, in Carole Reeves, ed., *A Cultural History of the Human Body in the Enlightenment* (Oxford: Oxford University Press, 2010), 15; Evans, *Unfortunate Objects*, 146.

9 Only one midwife’s will survives in the probate records held by the National Library of Wales, which is the will of Frances Huges of Haverfordwest NLW SD/1700/56.

10 NLW SD/LL/SM 2, 5, 8, 9, 11, 12, 15, 17, 19; B/SM/1.

11 142 records relating to infanticide cases are listed in the database, but some of these cases have more than one accused, or are individuals who were indicted more than once. The records used here are: NLW 4/46/2 (Catherine Roberts); NLW 4/47/7 (Mary Philips); NLW 4/58/2 (Elizabeth Bellis); NLW 4/62/1 (Sarah Owen); NLW 4/298/4 (Elizabeth Davies); 4/380/6 (Gwenllian David); NLW 4/530/6 (Mary Powell); 4/752/4 (Hannah John); NLW 4/900/3 (Ann Hughes); 4/906/4 (Maria Morris); NLW 4/1002/4 (Jane Griffith); NLW 4/1007/1 (Elizabeth Williams).

12 Powys County Archives (PCA) M/QS/SR 50-99).

13 Flintshire Records Office (FRO): Hawarden (D/BJ/324-328; Denbighshire Records Office (DRO): Llanarmon Dyffrin Ceiriog (PD/41); Llanarmon-yn-Ial (PD/43); Llandegla (PD/45); PCA Montgomeryshire: Aberhafesp (M/EP/2); Berriew (M/EP/3); Betws Cedwain (M/EP/4); Carno (M/EP/6); Castell Caereinion (M/EP/7); Guilsfield (M/EP/19); Kerry (M/EP/8); Llandinam (M/EP/15); Llanfihangel-yng-Ngwynfa (M/EP/21); Llangadfan (M/EP/24); Llangynyw (M/EP/27); Llanllwchaearn (M/EP/30); Llanwnnog (M/EP/38); Manafon (M/EP/40); Meifod (M/EP/41); Trefeglwys (M/EP/50); Tregynon (M/EP/51); Radnorshire: Llanbister (R/EP/1); Llandrindod (R/EP/47).

14 Evans, *Unfortunate Objects*, 145.

15 Donnison, *Midwives*, 38.

16 Samuel S. Thomas, ‘Early Modern Midwives: Splitting the Profession, Connecting the History’, *Journal of Social History,* 2009, 43, 116.

17 David Harley, ‘Provincial Midwives in England: Lancashire and Cheshire, 1660–1760’, in Hilary Marland, ed., *The Art of Midwifery: European Midwives in Europe* (London: Routledge, 1993), 28.

18
*Ibid*., 28.

19 Margaret Pelling, *The Common Lot: Sickness, Medical Occupations and the Urban Poor in Early Modern England* (Harlow: Longman, 1998), 203–29.

20 Adrian Wilson, *Ritual and Conflict: The social relations of childbirth in early modern England* (Farnham: Ashgate, 2013), 160.

21 NLW SD/LL/SM/8: O; SD/LL/SM/9:O; SD/LL/SM/11:O; SD/LL/SM/15:O; SD/LL/SM/19:O.

22 Evenden, *Midwives*, 54–9.

23 Cody, *Birthing the Nation*, 35; Elaine Hobby, ‘Introduction’ in Jane Sharp, *Midwives Book*, *or, The Whole Art of Midwifry Discovered*, ed. Elaine Hobby, Women Writers in English (Oxford: Oxford Univeristy Press, 1999), xiv.

24 Wilson, *Ritual and Conflict*, 160–1; Thomas, ‘Early Modern Midwives’, 116.

25 Thomas, ‘Early Modern Midwives’, 116; Evenden, *Midwives*, 38.

26 Alun Withey, *Physick and the Family: Health, Medicine, and Care in Wales, 160**0–**1750* (Manchester: Manchester University Press, 2011), 153.

27 The six married women were Elinor Ajax (NLW/SD/LL/SM/2); Margaret David (NLW/SD/LL/SM/5); Wenllian Harry (NLW/SD/LL/SM/8); Mary Hopkin (NLW/SD/LL/SM/9); Jane Llewellin (NLW/SD/LL/SM/12); Jane Morgan (NLW/SD/LL/SM/15); Jane Robert (NLW/SD/LL/SM/17); the one widow was Mary Winn (NLW/SD/LL/SM/19); the midwife for whom no marital status was given: Jane Jones (NLW/SD/LL/SM/11).

28 Wilson, *Making of Man-Midwifery*, 30; Cody, *Birthing the Nation*, 30; Williams, ‘Experience of Pregnancy’, 20, 67–86, fn 33.

29 Laura Gowing, ‘Ordering the Body: Illegitimacy and Female Authority in Seventeenth-Century England’ in Michael J. Braddick and John Walter, eds, *Negotiating Power in Early Modern Society: Order, Hierarchy and Subordination in Britain and Ireland* (Cambridge: Cambridge University Press, 2001), 49.

30 Withey, *Physick and the Family*, 158.

31 Thomas, ‘Early Modern Midwives’, 116.

32 NLW/SD/LL/SM/8.

33 NLW/SD/LL/SM/15.

34 NLW/SD/LL/SM/19 ii.

35 NLW/SD/LL/SM/19 ii.

36 Harley, ‘Provincial Midwives’, 39.

37 Wilson, *Making of Man-Midwifery*, 32.

38
*Ibid.*, 30; NLW/B/SM/1.

39 NLW/SD/LL/SM/2, NLW/SD/LL/SM/5, NLW/SD/LL/SM/19.

40 NLW B/CC/C(P)/39.

41 NLW B/CC/C(P)/39.

42 NLW/4/519/6.

43 NLW 4/752/4 (Hannah John).

44 NLW 4/298/4 (Elizabeth Davies).

45 NLW 4/380/6 (Gwenllian David).

46 NLW 4/46/2 (Catherine Roberts) and NLW 4/47/7 (Mary Philips).

47 NLW 4/1002/4 (Jane Griffith).

48 Gowing, *Common Bodies*, 159.

49 David Harley, ‘Historians as Demonologists: The Myth of the Midwife-Witch’, *The Society for the Social History of Medicine*, 1990, 3, 11–12.

50 NLW 4/380/6 (Gwenllian David).

51 PCA M/EP/4/O/RT/2; PCA M/EP/3/V/VM/3; FRO D/BJ/326.

52 NLW SD/1700/56. At the time of her death she lived in a five-room house consisting of two bed chambers, a garret, a small parlour and a kitchen, but her goods were valued at £12. To put this in perspective, Lesley Davison’s study of 56 spinster wills from the Diocese of St David’s between 1700 and 1715 revealed a range of inventory values from as little as 2d to as much as £322, with many under £20, and eighteen with £10 or less. A large portion of these women were rural householders able to maintain themselves through subsistence farming; however, most lived in much smaller houses. It would appear that Frances had a substantial house with a modest but comfortable level of wealth. See Davison, ‘Spinsters’.

53 NLW SD/LL/SM/19 ii.

54 NLW 6/629/2.

55 Wilson, *Ritual and Conflict*, 161.

56 NLW SD/LL/SM/8.

57 Gowing, *Common Bodies*, 159; Wilson, *Making of Man-Midwifery*, 26.

58 Source: PCA Berriew, M/EP/3/V/VM/4; Bettws Cedwain, M/EP/4/O/RT/1 & 2; Castell Caereinion, M/EP/7/O/RT/2& 3; Ceri, M/EP/8/O/RT/3, 5, 6, & 9; Llanarmon Dyffrin Ceiriog, PD/41/1/6 Meifod M/EP/41/O/RT/3, Trefeglwys M/EP/50/O/RT/3, Tregynon M/EP/51/O/RT/3.

59 Adrian Wilson, ‘The Ceremony of Childbirth and its Interpretation’ in Valerie Fildes (ed.) *Women as Mothers in Pre-Industrial Britain*, 2013 edn (Abingdon: Routledge, 1990), 62–73. See also Wilson, Ritual and Conflict, 153–210.

60 Frank T. Jones, ‘Notes on the Parish Registers of Merthyr Tydfil from AD 1703–1763’, *Y Cymmrodor the magazine of the Honourable Society of Cymmrodorion*, 1925, 35, 164.

61 Wilson, *Making of Man-Midwifery*, 34.

62
*Ibid.*

63 PCA M/EP/4/O/RT/1 & 2.

64 PCA M/EP/4/O/RT/1 & 2.

65 Wilson, ‘The Ceremony of Childbirth’, 72–3; Evenden, *Midwives*, 127.

66 Wilson, *Making of Man-Midwifery*, 15.

67
*Ibid.* 11; Roger Schofield, ‘Did the Mothers Really Die? Three Centuries of Maternal Mortality in “the World We Have Lost”’, in Lloyd Bonfield, Richard M. Smith and Keith Wrightson, eds, *The World We Have Gained: Histories of Population and Social Structure* (Oxford: Blackwell, 1986), 231–2; Irvine Louden, ‘Deaths in Childbed from the Eighteenth Century to 1935’ *Medical History,* 1986, 30, 22–4.

68 Wilson, *Making of Man-Midwifery*, 11–12.

69
*Ibid*.

70
*Ibid*.

71 PCA M/EP/3/V/VM/4; Montgomeryshire Genealogical Society (MGS) MR/C/27/1794; MGS MR/PR/45/1794.

72 PCA M/EP/7/O/RT/3.

73
*Ibid.*

74 MGS MR/C/15 1797.

75 Wilson, *Making of Man-Midwifery*, 1.

76
*Ibid*., 2.

77
*Ibid*.; Donnison, *Midwives*, 38;

78 King, ‘Midwifery’, 111.

79 NLW 4/1007/1 (Elizabeth Williams).

80 NLW 4/58/2 (Elizabeth Bellis); NLW 4/900/3 (Ann Hughes); 4/906/4 (Maria Morris).

81 Penelope J Corfield, *Power and the Professions in Britain, 1700**–**1850* (London: Routledge, 1995), 146.

82 Alannah Tomkins, ‘The Registers of a Provincial Man-Midwife, Thomas Higgins of Wem, 1781–1803’ *Shropshire Historical Documents, A Miscellany*, 2000, 4, 74.

83 PCA M/EP/41/W/AC/6.

84 PCA M/EP/21/O/RT/1.

85 Evenden, *Midwives*, 101; Josephine M. Lloyd, ‘The “Languid Child” and the Eighteenth-Century Man-Midwife’ *Bulletin of the History of Medicine*, 2001, 73, 641–79; Louden, ‘Deaths in Childbed’, 6.

86 King, ‘Midwifery’, 117.

87 NLW 4/752/4 (Hannah John).

88 Wilson, ‘Ceremony of Childbirth’; Gowing, *Common Bodies*, 149–50.

89 Gowing, *Common Bodies*, 158.

90
*Ibid.* 150; Alan Macfarlane, ed., *The Diary of Ralph Josselin 1616**–**1683* (London: Oxford University Press/British Academy, 1976) cited in Wilson, *Ritual and Conflict*, 1.

91 Wilson, *Ritual and Conflict*; Wilson, ‘Ceremony of Childbirth’.

92 Gowing, *Common Bodies*, 176.

93 Wilson, ‘Ceremony of Childbirth’; Gowing, *Common Bodies*, 149–50.

94
*Ibid.* 80–1.

95
*Ibid.*; Wilson, *Ritual and Conflict*, 179.

96 Gowing, *Common Bodies*, 156–76.

97 Keith Snell, *Parish and Belonging: Community, Identity and Welfare in England and Wales* (Cambridge: Cambridge University Press, 2006), 85.

98
*Ibid.* 86.

99 PCA M/EP/50/O/RT/1.

100 PCA M/EP/8/O/RT/6.

101 Gowing, *Common Bodies*, 157.

102 PCA M/EP/40/O/RT/1.

103 PCA M/QS/1750-1799 Session Rolls (SR 50E27).

104 Gowing, ‘Ordering the Body’, 43–62.

105 Nick Woodward, ‘Infanticide in Wales, 1730–1830’, *Welsh History Review*, 2007, 23, 110; NLW 4/530/6 (Mary Powell).

106 NLW 4/62/1 (Sarah Owen).

107 PCA M/EP/15/O/RT/1.

108 PCA M/EP/50/O/RT/2.

109 Wilson, *Ritual and Conflict*, 180.

110 Gowing, *Common Bodies*, 156.

111 PCA M/EP/4/O/RT/2.

112 Wilson, ‘Ceremony of Childbirth’, 80–1.

113 PCA M/EP/3/V/VM/3.

114 PCA M/EP/24/O/RT/1; PCA M/EP/41/V/VM/1.

115 ‘Wilson, ‘Ceremony of Childbirth’, 80–1.

116 PCA M/EP/4/O/RT/1 (payment by officials in Bettws Cedewain for a midwife and rent for the wife of William Jones, a soldier in 1762); PCA M/EP/41/O/RT/3 (payment by officials in Meifod for a midwife and rent for the wife of Thomas Jones in the 1780s).

117 Gowing, ‘Ordering the Body’, 43–62.

118 Gowing, *Common Bodies*, 154.

119 Cody, *Birthing the Nation*, 36.

120 Gowing, *Common Bodies*, 154; Abby Chandler, ‘From Birthing Chamber to Court Room: The Medical and Legal Communities of the Colonial Essex County Midwife’, *Early Modern Women: An Interdisciplinary* Journal, 2015, 9, 110; Cody, *Birthing the Nation*, 36; Crawford ‘Construction’, 21; Wilson, *Making of Man-Midwifery*, 1.

121 Gowing, *Common Bodies*, 153.

122 PCA M/EP/4/O/RT/2.

123 PCA M/EP/7/O/RT/3.

124 PCA M/EP/19/O/RT/1.

125 Cody, *Birthing the Nation*, 36.

126 Gowing, *Common Bodies*, 159.

127
*Ibid.* 160; Crawford, ‘Construction’, 21.

128 Manchester Records Office (MRO) GB124.E4/28/4/5. See also, documents 8, 9, 19, 19 and 50 in the same folder.

129 Wilson, *Ritual and Conflict*, 26.

130
*Ibid*.

131 NLW SD/LL/SM/8 Wenllian Harry.

132 Wilson, *Ritual and Conflict*, 27.

133 Source: PCA Llandegley R/EP/1/O/BB/29/49; Berriew M/EP/3/O/BB/1/73; Bettws Cedewain M/EP/14/O/BB/4/12; Llandinam M/EP/15/O/BB 1/95 & M/EP/15/O/X/1; Llangynyw M/EP/27/O/BB/1/12 Trefeglwys M/EP/50/O/BB1/21.

134 Muir, ‘Courtship, Sex and Poverty’, 77–8.

135
*Ibid*., 63–4; Angela Muir, ‘Illegitimacy in Eighteenth-Century Wales’, *Welsh History Review*, 2013, 23, 351–5.

136 Gowing, *Common Bodies*, 176; Wilson, ‘Ceremony of Childbirth’; Williams, ‘Experience of Pregnancy’, 79.

137 Williams, ‘Experience of Pregnancy’, 69.

138 DRO PD/41/1/6.

139 PCA M/EP/4/O/RT/7; PCA M/EP/19/O/RT/1.

140 PCA M/EP/7/O/RT/3; M/EP/19/O/RT/1.

141 PCA R/EP/47/W/AC/1.

142 PCA M/EP/41/V/VM/1.

143 Linda A. Pollock, ‘Embarking on a Rough Passage: The Experience of Pregnancy in Early-Modern Society’ in Fildes, *Women as Mothers*, 49.

## Notes

Angela Muir was appointed Lecturer in British Social and Cultural History in the Centre for English Local History at the University of Leicester in 2018. She was the 2017–18 EHS Power Fellow and Lecturer in Welsh History at Cardiff University. She completed her doctoral research at the University of Exeter with funding from the Wellcome Trust and SSHRC. Her research looks at the experience and broader social, cultural and medical context of illegitimacy and childbirth in eighteenth-century Britain.

